# A New Method to Address the Importance of Detoxified Enzyme in Insecticide Resistance – Meta-Analysis

**DOI:** 10.3389/fphys.2022.818531

**Published:** 2022-03-02

**Authors:** Yifan Wang, Alan E. Wilson, Nannan Liu

**Affiliations:** ^1^Department of Entomology and Plant Pathology, School of Agriculture, Auburn University, Auburn, AL, United States; ^2^School of Fisheries, Aquaculture and Aquatic Sciences, Auburn University, Auburn, AL, United States

**Keywords:** insecticide resistance, metabolic detoxification genes, diverse insect species, meta-analysis, overexpression of detoxification genes

## Abstract

Insect-borne diseases, such as malaria, and plant pathogens, like the tobacco mosaic virus, are responsible for human deaths and poor crop yields in communities around the world. The use of insecticides has been one of the major tools in pest control. However, the development of insecticide resistance has been a major problem in the control of insect pest populations that threaten the health of both humans and plants. The overexpression of detoxification genes is thought to be one of the major mechanisms through which pests develop resistance to insecticides. Hundreds of research papers have explored how overexpressed detoxification genes increase the resistance status of insects to an insecticide in recent years. This study is, for the first time, a synthesis of these resistance and gene expression data aimed at (1) setting up an example for the application of meta-analysis in the investigation of the mechanisms of insecticide resistance and (2) seeking to determine if the overexpression detoxification genes are responsible for insecticide resistance in insect pests in general. A strong correlation of increased levels of insecticide resistance has been observed in tested insects with cytochrome P450 (CYP), glutathione-S-transferase (GST), and esterase gene superfamilies, confirming that the overexpression of detoxification genes is indeed involved in the insecticide resistance through the increased metabolism of insecticides of insects, including medically (e.g., mosquito and housefly) and agriculturally (e.g., planthopper and caterpillar) important insects.

## Introduction

There are thought to be approximately five million insect species worldwide with ∼80% remaining to be discovered (Stork, 2018). Among these, agricultural (e.g., whitefly, armyworm, and grasshopper) and medical (e.g., mosquito, housefly, and cockroach) pests can have a significant negative impact on food supplies and human health. Globally, insect pests are responsible for around 35% of potential crop yield losses (Popp et al., 2013; Mesterházy et al., 2020). Besides damage and monetary loss, the transmission of plant viruses by insect vectors can also reduce crop yield and quality (Fereres and Raccah, 2015). Likewise, medically important pests can transfer lethal pathogens capable of compromising human life (e.g., malaria, yellow fever, and dengue, all of which are transferred by mosquitoes) and impose huge socio-economic burdens on the affected populations (Bhatt et al., 2013; Kuehn, 2018; Shaw and Catteruccia, 2019). For instance, the housefly is capable of transmitting more than 100 human and animal diseases, including cholera, typhoid fever, salmonellosis, and polio (Ostrolenk and Welch, 1942; Feng et al., 2018). Common pest management strategies include installing traps to monitor and control the density of pests (Baldacchino et al., 2015), rearing natural enemies to prey on the pests (Lazaro et al., 2015), or spraying insecticide. All these methods are routinely employed to control pest populations in an attempt to avoid production and monetary losses, insect-borne disease outbreaks, and other associated impacts.

Of these various pest management strategies, the use of insecticides is still the most widely-used approach [i.e., roughly two million tons of pesticides being sprayed on insect pests in communities around the world every year (Sharma et al., 2019)] because of its immediate and rapid reduction of pest populations (Strode et al., 2012). However, this widespread overuse of insecticides has led to the emergence of new problems. The first is that insecticide resistance is rapidly becoming a serious obstacle to the effective pesticide control of many pests. Moreover, and there are only a very limited number of new insecticides being commercialized for vector control (Liu, 2015). Because of the frequent and long-term use of insecticides, pests that carry a resistance gene can survive when exposed to an insecticide and these genes can be inherited in the subsequent generations, thus leading to the widespread development of insecticide resistance in local populations (Liu, 2015; Smith et al., 2016). Crucially, the greater the resistance ability of the pest to a particular insecticide, and hence the survivability of the pest when exposed, the greater the associated crop loss or the transmission of insect-borne diseases (Atyame et al., 2019; Chen et al., 2021). The phenomenon of insecticide resistance was first recognized by Dr. Melander in 1914 with sulfur lime (Melander, 1914). The resistance of both medical and agricultural pests to a variety of insecticides has now been reported in pest populations worldwide (Naveen et al., 2017; Camara et al., 2018; Okia et al., 2018; Lopes et al., 2019).

Numerous papers have been published that address the underlying mechanisms that govern the development of insecticide resistance. Some have shown evidence to suggest that gene mutations can decrease the sensitivity of the target site and thus be responsible for increasing insecticide resistance (Dong, 2007; Xu et al., 2011; Tmimi et al., 2018; Zeidabadinezhad et al., 2019; Yavaşoglu et al., 2021), while others have argued that the overexpression of the detoxification gene is the most important mechanism driving the development of insecticide resistance (Xu et al., 2016; Feng et al., 2018; Jin et al., 2019). It is generally agreed, however, that target site insensitivity and increased insecticide metabolism are both involved. Consequently, these two mechanisms have been intensively studied (Antonio-Nkondjio et al., 2011; Liu, 2015; Dang et al., 2017). Hundreds of papers have explored how overexpressed genes increase the resistance status of insects to insecticides in recent years. This study, for the first time, quantitatively synthesizes these data using meta-analysis to provide an overview of the impact of the overexpression of genes in diverse insect species against different insecticides as well as identify the gene families and clades responsible for the most widely observed examples of this overexpression. This study could be used as an example to inspire more researchers to use meta-analysis to answer more questions.

## Materials and Methods

### Literature Search Strategy

**FIGURE 1:**
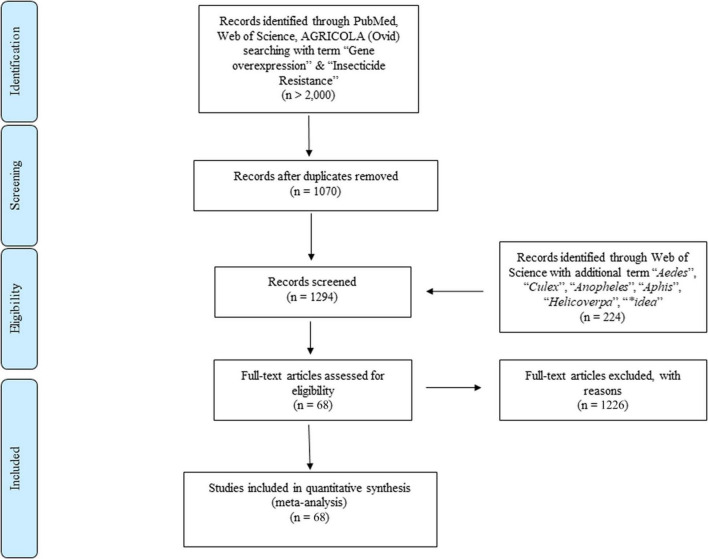
Flow diagram based on PRISMA.

To fully understand the metabolic detoxification mechanisms conferred by the gene overexpression that are importantly involved in the insecticide resistance development in general, we quantitatively compared the gene expression between resistant and susceptible strains and its correlation by conducting, a literature search in Web of Science, PubMed, and AGRICOLA (Ovid) using the following search terms: “gene overexpression” AND “insecticide resistance” OR “pesticide resistance.” In addition, “*Culex*” OR “*Aedes*” OR “*Anopheles*” OR “*Aphid*” OR “*Helicoverpa*” OR “*Spodoptera*” OR “*idae” (Sampson et al., 2009) were used in conjunction with “gene overexpression” AND “insecticide resistance” in Web of Science. The Boolean truncation (*) was used to capture all variations of a search term. A schematic outline of the search strategy is illustrated in Figure 1. A publication date range of 2000 to 2020 was used and although no restrictions were placed on the country of publication, only reports in English were included; a study was included only if it was an original journal article (books, review articles, and letters to the editor were excluded). The literature search was performed between January and March 2020 (Figure 1).

### Study Selection, Inclusion, and Exclusion Criteria

To be considered eligible for inclusion, a study had to meet the following criteria:

(1) Gene expression must have been measured through relative mRNA abundance by applying qPCR or real-time PCR. Protein-based or enzyme activity-based measurements were excluded because real-time PCR is use for measuring gene expression as well as validating Microarray and RNAseq data, thus, we only use RT-PCR data in this analysis. In addition, meta-analysis requires the similar measurement methods for the data examination and the mix of different gene expression testing methodology, such as mix of microarray and RNAseq and RT-PCR, may generate unexpected variation. Thus, we only chose RT-PCR as our methodology in the study. Moreover, all the genes should be annotated, the genes with Gene Bank ID or vector base ID or other type ID will be excluded. The gene expression should be measured on the level of the whole body.
(2) A study must have contained a susceptible strain (or susceptible field strain) as the control and a resistant strain (or resistance field strain) as the measurement of comparison. Thus, studies measuring the gene expression level of insects collected from different locations that did not consider significant differences in the resistance levels of insects from different populations were excluded. The studies that measure the gene expression level before and after insecticides exposure were excluded.
(3) A study must have reported either the mean value of the gene expression level and some estimate of error (e.g., standard deviation, standard error, or 95% confidence interval) around the mean estimates for the susceptible and resistant strains or the ratio of these gene expression levels.
(4) A study must have reported no gene modification of the strains. Thus, studies testing gene overexpression after editing the genomes of the strains using RNAi, CRISPR-Cas9, or other gene-editing methods were excluded.
(5) A study must have reported no transformation of the gene to other materials; studies testing gene overexpression after the gene had been transferred to sf9 cell, *Drosophila*, or some other materials were excluded.

### Data Extraction and Analyses

To gather data from each study, a standardized form, including author(s), publication year(s), insecticide(s), and species, was utilized that facilitated the extraction of the important study characteristics. To perform moderator analysis, the class of insecticides, insect genus, and gene name were collected from each study. The sample size, mean value, and variance components (e.g., standard deviation, standard error, or 95% confidence interval) of gene expression were also collected. Where necessary, standard errors or 95% confidence intervals were converted to standard deviation using sample sizes and t-statistics. Data from the figures were extracted using R with the *metaDigitise* package (1.0.0) (Pick et al., 2019). For figures that presented linear gene expression data on a log_10_ transformed scale, the data collected from each figure were estimated by raising each data point by 10. Because of scaling constraints, the error presented in the figures was estimated after recreating the original figures with estimated data and then adjusting the error bars until they were similar to those shown in the original figure. Only 30 observations (4.2% of all data) were collected using this method.

### Effect Size Calculation and Data Analyses

For this study, the effect size reflected the strength of the relationship between gene expression and insecticide resistance (Kelley and Preacher, 2012; Schäfer and Schwarz, 2019). This effect size was calculated for each experiment in a study by comparing the relative gene expression values between the control (susceptible) and the treatment (resistant) strains. The effect size metric used in this study was the natural log response ratio [LnRR; (Hedges et al., 1999)], which is calculated as the natural log-transformed ratio of mean expression values between the treatment (resistant) and control (susceptible) (Havird et al., 2013). However, to more accurately estimate the magnitude of gene expression involved in insecticide resistance, weighted effect sizes that include the variance of LnRR are needed.

For the studies that only provided the relative expression ratio (i.e., the gene expression ratio between resistant strain and susceptible strain) but did not provide the exact gene expression data for both strains (Bariami et al., 2012; Cifuentes et al., 2012; Feng et al., 2018), LnRR was calculated by directly taking the natural log of the relative expression ratio, and the variance was assigned a value of 0.1. For the studies that provided a mean and error for gene expression for both susceptible and resistance strains (Zhang et al., 2012; Jin et al., 2019; Ma et al., 2019), the LnRR effect size and variance was calculated through R using the “metafor” package (Viechtbauer, 2010). An effect size (and lower 95% CI) > 0 indicates that the detoxification gene is overexpressed in the resistant strain compared to the susceptible strain and thus provides evidence that gene overexpression is indeed one of the mechanisms involved in the development of insecticide resistance. On the contrary, the overexpression of the detoxification gene may not be involved in the mechanism of insecticide resistance. To facilitate the interpretation of the natural log-transformed effect sizes, each was converted back to a linear ratio scale. For example, an LnRR effect size of 0.41 would represent a 1.5 fold overexpression of a detoxification gene in a resistant strain (calculated as exp^0.41^ = 1.5, 1.5/1 = 1.5) compared to the susceptible strain.

Since our dataset included some studies where error estimates were not provided with mean gene expression, two separate analyses were conducted to evaluate the robustness of our findings. The first analysis was an unweighted analysis that included mean gene expression for all of the studies. The second analysis was a weighted analysis, which only included studies that provided the mean gene expression data and the associated error for both resistant and susceptible strains. Both of the two analyses were conducted using a random-effects model for the gene expression of the susceptible and resistant strain in R using both the “metafor” (Viechtbauer, 2010) and “meta” (Balduzzi et al., 2019) packages. The statistical heterogeneity, i.e., the magnitude of the changes of the effect sizes in this study, of the treatment effect among studies was assessed using the inconsistency (I^2^) test, in which values above 30 and 50% were considered indicative of moderate and high heterogeneity, respectively. To improve the quality of our results, we conducted sensitivity analyses by omitting each study in turn and re-calculating the effect size to check the resulting difference in the effect size.

### Moderator Analysis

We classified genes into different gene families and clades and insecticides into different classes according to the modes of action recommended by the Insecticide Resistance Action Committee (IRAC) (Sparks et al., 2020). Insect species were grouped according to their genus that is classified as a medical vector or agricultural pest. Medical vectors represent genera that threaten human health; for example, insects in the genus *Aedes* transmit the dengue virus to humans. Agricultural pests include genera that threaten plant health such as *Helicoverpa*, which can damage cotton and tomato crops. This made it possible to compare the effect on insecticide resistance of overexpressed genes in different gene families and different P450 gene clades, as well as different insecticide classes, different genera, and different vector types. Finally, moderator analyses were conducted to compare the effect on insecticide resistance of overexpressed genes in different gene families within specific medical or agricultural pests. All moderator analyses were conducted utilizing unweighted meta-analyses for all available effect sizes.

### Publication Bias and Sensitivity Analysis

Publication bias, i.e., the bias of the authors, editors, and/or reviewers to the publication of data, was evaluated by visually analyzing funnel plots (*metaviz* R package), Egger’s tests (*dmetar* R package), and regression tests and p-curves (*metafor* R package) (Song et al., 2010). The sensitivity analysis, i.e., a method used to test whether the results will be changed by a certain paper, was performed using the “leave1out” analysis in the *metafor* R package (Saltelli et al., 2008). GOSH (Graphic Display of Study Heterogeneity) plot was performed in the *metafor* R package (Olkin et al., 2012).

## Results

### Overall Dataset

All data used in this study were provided in the online Supplementary Material for transparency and future analyses (Supplementary Table 1). Based on our search strategy and inclusion criteria, 68 published papers were selected and analyzed in this meta-analysis containing a total of 14 types of insecticides, the three major classes of insecticides are neonicotinoids (13 papers), organophosphates (7 papers), and pyrethroids (31 papers). Three major gene families, P450 (58 papers), Esterase (11 papers), and GST (16 papers) with over 200 genes and CYP4 (35 papers), CYP6 (50 papers), CYP9 (16 papers), and over 40 gene clades were included in the selected papers. The data distribution includes 22 insect genera (in 68 papers) that can transmit pathogens to either humans (medical vectors) or plants (agricultural pests) (Table 1). The remaining papers were excluded as they failed to meet one or more of the inclusion criteria. Note that all effect sizes below are presented in the form of back-transformed linear scale ratios to facilitate the interpretation of the results (Figure 1).

**TABLE 1:** Number of studies in different categories.

		# Papers			# Papers
Total		68	P450 Gene Clade	cyp4	35
Insecticide Classes	Avermectins	2		cyp417	7
				cyp425	6
	Buprofezin	2		cyp439	8
	Carbamates	1		cyp6	50
	DDT	2		cyp418	6
	Diamides	1		cyp408	3
	Neonicotinoids	13		cyp427	4
	Organophosphate	7		cyp18	6
	Organochlorines	1		cyp304	9
	Pyrethroid	31		cyp306	8
	Phenlypyrazoles	2		cyp303	10
	Spinosyns	1		cyp15	3
	Sulfoxaflor	1		cyp305	9
	Tetronic	1		cyp419	5
	Mixture of insecticides	5		cyp302	5
				cyp301	7
Vector Type	Agricultural Vector*	44		cyp314	7
	Medical Vector**	24		cyp404	4
Gene Family	P450	58		cyp9	16
	Esterase	11		cyp321	1
	GST	16		cyp337	4
P450 Gene Clade	CYP4	35		cyp332	2
	CYP6	50		cyp353	8
	CYP9	16		cyp426	2
	Others	27		cyp307	3
Insect Genera	*Aedes*	4		cyp315	5
	*Amsacta*	1		cyp380	5
	*Anopheles*	8		cyp340	1
	*Aphis*	6		cyp3085	1
	*Apolygus*	1		cyp3092	1
	*Bemisia*	2		cyp2	1
	*Blattella*	1		cyp3323	1
	*Ceratitis*	1		cyp49	3
	*Culex*	5		cyp3	1
	*Drosophila*	1		cyp5	1
	*Diaphorine*	1		cyp12	2
	*Frankliniella*	1		cyp319	1
	*Helicoverpa*	4		cyp325	1
	*Laodelphax*	9		cyp3115	1
	*Leptinotarsa*	2			
	*Musca*	5			
	*Nilaparvata*	6			
	*Plutella*	6			
	*Rhopalosiphum*	1			
	*Sogatella*	1			
	*Spodoptera*	1			
	*Triatoma*	1			

**There are 20 papers, nine papers and four papers investigate about the gene expression of P450, GST and Esterase in medical vector respectively. *There are 38 papers, seven papers, and seven papers investigate about the gene expression of P450, GST and Esterase in agricultural vector respectively.

### Effect Size Summary

The linear scaled effect sizes (i.e., the number used to reflect the strength of the relationship between the two variables statistically) of the unweighted and weighted analyses (including studies both with and without susceptible strain) were 1.82 (95% confidence interval: 1.65–2.00, *P*-value < 0.01) and 1.50 (95% confidence interval: 1.35–1.67, *P*-value < 0.01), respectively. Overall, the expression level of detoxification genes tested was over 1.5 that seen in the resistant strains compared to the susceptible strains, suggesting that the overexpression of detoxifying genes is indeed associated with increased insecticide resistance (Supplementary Table 2).

**FIGURE 2:**
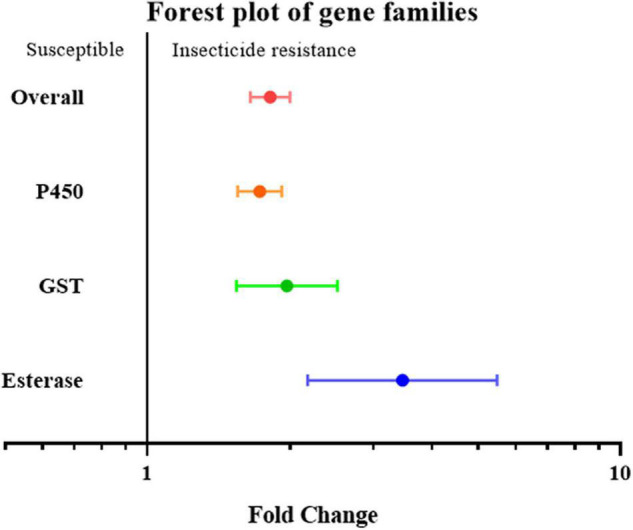
Meta-analytical, sub-group estimate of gene expression related to insecticide resistance, with 95% CI in the P450, GST, and esterase gene families. The overall effect represents the collective effect of all the studies in terms of their unweighted analyses.

**FIGURE 3:**
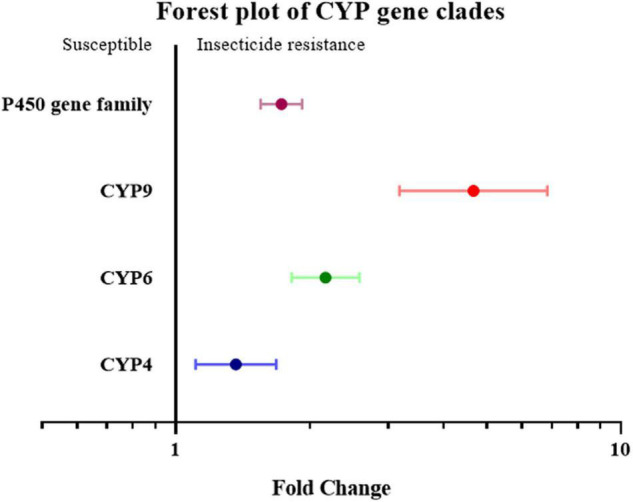
Meta-analytical, sub-group estimate of gene expression related to insecticide resistance, with 95% CI in the P450 gene families classified by gene clade. The overall effect represents the collective effect of all P450 studies.

To further investigate overexpression of which gene family or clade is responsible for increased insecticide resistance, the overexpression of cytochrome P450, esterase, and GST (glutathione S-transferase) was analyzed. Given the small number of studies on the overexpression of esterase genes, all esterase-related genes, including carboxylesterase (COE) and acetylcholinesterase (AchE), were classified simply as the “esterase” gene. None of the effect sizes for these three gene families overlapped with the null group: the expression level of the esterase gene was 3.46 fold (*P*-value < 0.01) higher in resistant strain compared to the susceptible strain; the expression level of the GST gene was 1.97 fold (*P*-value < 0.01) higher in resistant strain compared to susceptible strain; and the expression level of the P450 gene was 1.73 fold (*P*-value < 0.01) higher in resistant strain compared to susceptible strain. All three gene families were clearly involved in insecticide resistance through upregulated gene expression (Figure 2). This abundance of studies made it possible to further split the P450 gene group into different clades, in which the expression level of the CYP4, CYP6, and CYP9 genes in the resistant strains were further analyzed showing to be 1.37 fold (*P*-value < 0.01), 2.17 fold (*P*-value < 0.01), and 4.67 fold (*P*-value < 0.01), higher compared to the susceptible strains, respectively (Figure 3). In contrast, the gene expression for the rest of the clade did not significantly contribute to insecticide resistance, with the sole exception being CYP332, which exhibited a significant difference in just two studies (*P* < 0.01) (Brun-Barale et al., 2010; Xu et al., 2016; Supplementary Table 2). There was, however, a large proportion of heterogeneity in the overall summary effect size (I^2^ > 94%), suggesting that a single summary effect size may not be as informative. To further explore this heterogeneity, these data were further partitioned into various groups using specific moderators.

### Factors Contributing to Heterogeneity Between Studies

**FIGURE 4:**
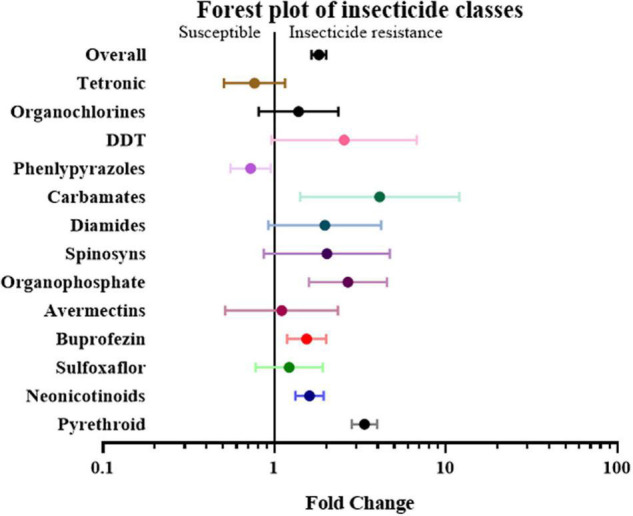
Meta-analytical, sub-group estimate of the gene expression related to insecticide resistance, with 95% CI across different insecticide classes. The overall effect represents the collective effect of all the relevant studies.

First, the papers and effect sizes were classified based on the type of insecticide used when measuring the resistance level of a specific insect strain. If a study involved more than two insecticides, it was classified as “mix-insecticide” (Elzaki et al., 2016; Goindin et al., 2017; Zhang et al., 2017; [Bibr B52][Bibr B53]). The detoxification genes in the strains that were resistant to buprofezin, carbamates, neonicotinoids, organophosphates, and pyrethroid were 1.54 fold (*P*-value < 0.01), 4.11 fold (*P*-value < 0.01), 1.60 fold (*P*-value < 0.01), 2.68 fold (*P*-value < 0.01) and 3.35 fold (*P*-value < 0.01) higher compared to the susceptible strain, respectively. In contrast, the overexpression of detoxification genes was not significantly influenced by the insect’s resistance to other classes of insecticides, such as Avermectins, DDT, Diamides, Organochlorines, Phenlypyrazoles, Spinosyns, Sulfoxaflor, and Tetronic (Figure 4 and Supplementary Table 2).

**FIGURE 5:**
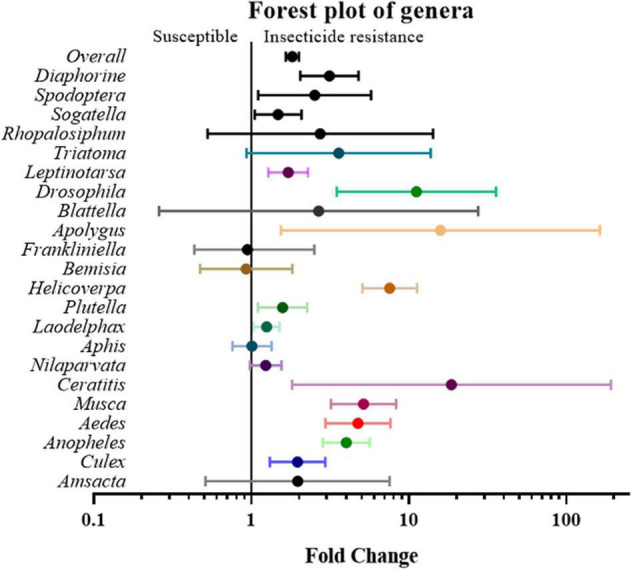
Meta-analytical, sub-group estimate of the gene expression related to insecticide resistance, with 95% CI in different insect genus and vector types. The overall effect represents the collective effect of all the relevant studies.

Next, the studies and their effect sizes were classified based on their target species to investigate whether the positive relationship between gene expression and insecticide resistance exists across species. The analysis revealed that *Aedes* (4.74 fold, *P* value < 0.01), *Anopheles* (3.99 fold, *P* value < 0.01), *Apolygus* (15.85 fold, *P* value < 0.05), *Ceratitis* (18.60 fold, *P* value < 0.05), *Culex* (1.96 fold, *P* value < 0.01), *Drosophila* (11.14 fold, *P* value < 0.01), *Diaphorine* (3.12 fold, *P* value < 0.01), *Helicoverpa* (7.55 fold *P* value < 0.01), *Laodelphax* (1.25 fold, *P* value < 0.05), *Leptinotarsa* (1.71 fold, *P* value < 0.01), *Musca* (5.15 fold, *P* value < 0.01), *Plutella* (1.58 fold, *P* value < 0.05), *Sogatella* (1.48 fold, *P* value < 0.05), and *Spodoptera* (2.52 fold, *P* value < 0.05) all showed significant differences between gene expression in susceptible and resistant strains, while other genera, such as *Amsacta*, *Aphis*, *Bemisia*, *Blattella*, *Frankliniella*, *Nilaparvata*, *Rhopalosiphum*, and *Triatoma*, did not (Figure 5 and Supplementary Table 2).

**FIGURE 6:**
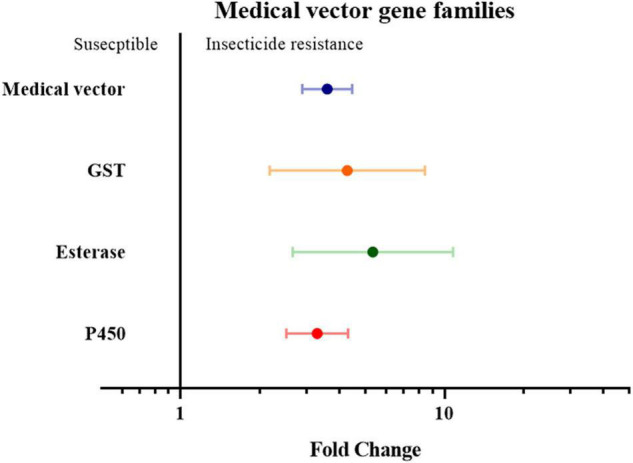
Meta-analytical, sub-group estimate of the gene expression related to insecticide resistance, with 95% CI in the medical vector with different gene families and the P450 gene clade. The overall effect represents the collective effect of all the studies investigating the medical vector.

**FIGURE 7:**
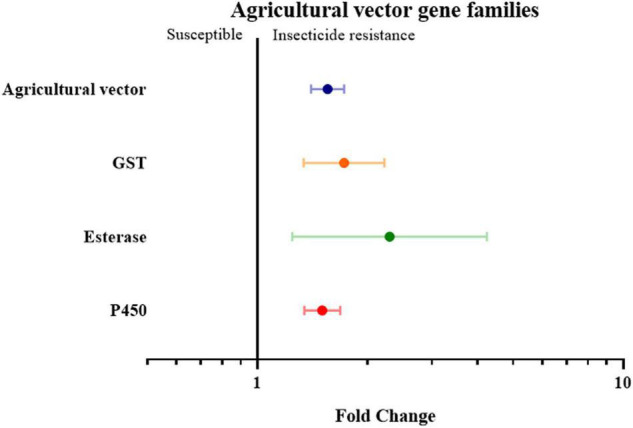
Meta-analytical, sub-group estimate of the gene expression related to insecticide resistance, with 95% CI in the agricultural vector with different gene families and the P450 gene clade. The overall effect represents the collective effect of each insecticide class in all the relevant studies.

Finally, the insect genera were grouped according to their role as medical or agricultural vectors. For both medical and agricultural vectors, the expression of detoxification genes in resistance strains are significantly higher than the susceptible strains (3.59 fold in medical vectors group with *P-*value < 0.01, and 1.55 fold in agricultural group with *P-*value < 0.01). To investigate which gene families are involved in insecticide resistance in both the agricultural pests group and medical vectors group, each group was further classified into three groups based on their gene families. In the medical vector group, the expression of detoxification genes in all three gene families was higher in the resistant strains than in the susceptible strains by 4.28 fold in the GST genes (*P*-value < 0.01), 5.35 fold in the esterase genes (*P*-value < 0.01), and 3.29 fold in the P450 genes (*P* value < 0.01), respectively (Figure 6 and Supplementary Table 2). Similarly, across the agricultural vectors, the expression levels of the GST genes, the esterase genes, and the P450 gene were 1.72 fold (*P*-value < 0.01), 2.30 fold (*P*-value < 0.01), and 1.50 fold (*P*-value < 0.01) higher.(Figure 7 and Supplementary Table 2).

### Publication Bias and Sensitivity Analysis

**FIGURE 8:**
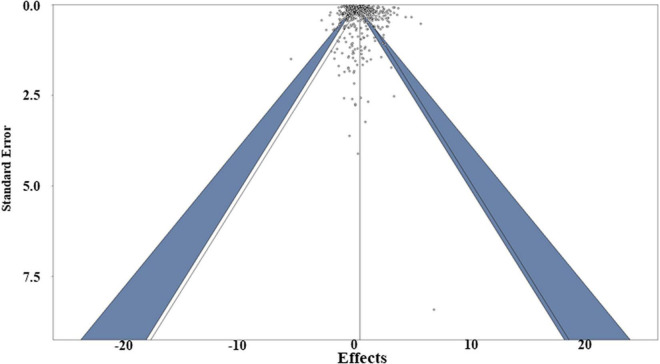
Funnel plot of the weighted meta-analysis of the studies, including both susceptible and resistant expression data. The dark blue area means represent the 95% confidence interval.

Publication bias (i.e., the bias about the direction or significance of the manuscripts from authors, editors and reviewers when they are submitting or accepting manuscripts) was assessed using multiple approaches, including funnel plot evaluations, regression and Egger’s tests (Egger et al., 1997). The Egger’s test revealed the funnel plot asymmetry with *P* value < 0.05, while the regression test of the funnel plot of the weighted meta-analysis showed symmetry (*P* = 0.23), (Figure 8), indicating there is no clear publication bias in this study. To further address the potential for publication bias, a P-curve analysis was used to check whether any studies showed false positives [i.e., findings that statistics suggest are meaningful when they are not (Simonsohn et al., 2014)]. The resulting curve was right-skewed with > 95% of the studies showing *p* < 0.01, suggesting truly significant *p-*values dominated the dataset (Supplementary Figure 1). A leave-one-out sensitivity analysis (i.e., the method used to examine whether the result will change significantly due to one certain paper) was also conducted for both weighted and unweighted meta-analyses to determine whether individual studies influenced the overall results. The sensitivity analysis showed that no single effect size significantly biased the overall effect size (Supplementary Tables 3, 4). Consequently, a Graphic Display of Study Heterogeneity (GOSH) plot was created to visually depict the heterogeneity, showing it to be equally distributed across the dataset (Supplementary Figure 2; Olkin et al., 2012).

## Discussion

We further confirmed that the overexpression of detoxified genes is indeed responsible for insecticide resistance through the rigorous analysis of data from 68 studies, including three major detoxification gene families (P450s, GSTs, and Esterases), various species (e.g., *Aedes*, *Anopheles*, *Plutella*, *Helicoverpa*, etc.), and different type of insecticides (e.g., permethrin, neonicotinoids, organophosphates, etc.). Plenty of studies have proven the importance of the overexpression of detoxified genes in insecticide resistance (Feyereisen, 1999; Enayati et al., 2005; Liu, 2015) and confirmed their large responsibility for enhancing the metabolic detoxification of insecticides (Plapp, 1976; Liu, 2015; Lu et al., 2020). There is also a review focusing on the *Spodoptera*, which also confirms the importance of the overexpression of detoxified genes in insecticide resistance (Amezian et al., 2021).

However, resistance was not associated with significant differences in gene expression for all insecticides. For example, traditional classes of insecticide (e.g., pyrethroid, organophosphate, carbamates, and etc.) exhibited significant differences, while some insecticides (e.g., diamides, avermectins, sulfoxaflor, and etc.) showed little difference between resistant and susceptible strains. As we have known, the long-term and overuse of an insecticide can lead to resistance due to diverse mechanisms (Liu, 2015). Thus, the insecticides that were not shown to have large differences in detoxification gene expression in resistant and susceptible strains maybe because they are not as widely used as traditional insecticides. So, even if resistance develops, resistance may be due to other mechanisms than the high expression of detoxification genes. Most species were found to be able to upregulate the expression of detoxification genes against insecticides, as most insecticides are broad-spectrum and are used to control multiple insect species (Ahmad et al., 2002; Sparks et al., 2020). Both medical and agricultural pests show the higher detoxified gene expression in resistance strain compared with susceptible strain. This proves that both medical and agricultural pests received similar insecticide pressure. Besides, all the detoxified gene families (GST, Esterase, P450) in the medical and agricultural pests are significantly overexpressed in resistance strains compared with susceptible strains.

This paper demonstrates a new method in the investigation of the mechanism of insecticide resistance. Based on this example, we conclude that the overexpression of detoxified gene is involved in insecticide resistance. The following researchers could focus on other different questions through meta-analysis, for example, what kind of fitness cost happened in resistance strain compared to the susceptible strain? Although there is no experiment be done in the meta-analysis, the result of meta-analysis is based on numerous experimental papers with the real experimental data. Otherwise, one experimental paper can only focus on one insect species to one or several insecticides with the expression of several detoxified genes. In the meta-analysis, we can combine and analyze the data from 22 insect genera to 14 types of insecticides with the expression of over 200 genes in a total of 68 papers.

## Data Availability Statement

The datasets presented in this study can be found in online repositories. The names of the repository/repositories and accession number(s) can be found in the article/Supplementary Material.

## Author Contributions

NL, YW, and AW conceived and designed the study, wrote the manuscript, and reviewed the manuscript. YW searched the manuscript and analyzed the data. All authors contributed to the article and approved the submitted version.

## Conflict of Interest

The authors declare that the research was conducted in the absence of any commercial or financial relationships that could be construed as a potential conflict of interest.

## Publisher’s Note

All claims expressed in this article are solely those of the authors and do not necessarily represent those of their affiliated organizations, or those of the publisher, the editors and the reviewers. Any product that may be evaluated in this article, or claim that may be made by its manufacturer, is not guaranteed or endorsed by the publisher.

## Supplementary Material

Supplementary Figure 1 P-curve of the weighted meta-analysis. The observed p-curve includes 372 statistically significant (p < 0.05) results, of which 364 are p < 0.025. There were 98 additional results entered but excluded from P-curve because they were P > 0.05.

Supplementary Figure 2 The graphic display of study heterogeneity.

Supplementary Table 1 List of specific summary and data for each paper in meta-analyses.

Supplementary Table 2 Effect sizes for subgroups.

Supplementary Table 3 Sensitivity analysis for the unweighted meta-analysis.

Supplementary Table 4 Sensitivity analysis for the weighted meta-analysis.

Supplementary Data 5 R code for meta-analyses.
