# Supplementary figures and images for: A New Method to Address the Importance of Detoxified Enzyme in Insecticide Resistance – Meta-Analysis
Source: Front Physiol. 2022 Mar 2;13:818531. doi: 10.3389/fphys.2022.818531 (PMC8924616; doi:10.3389/fphys.2022.818531)

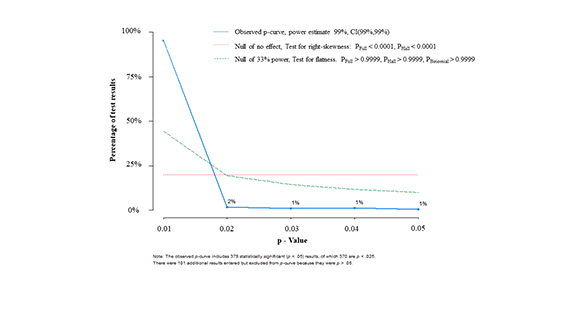

Supplement: Supplementary Figure 1 — P-curve of the weighted meta-analysis. The observed p-curve includes 372 statistically significant (p < 0.05) results, of which 364 are p < 0.025. There were 98 additional results entered but excluded from P-curve because they were P > 0.05. [file Image_1.TIF]

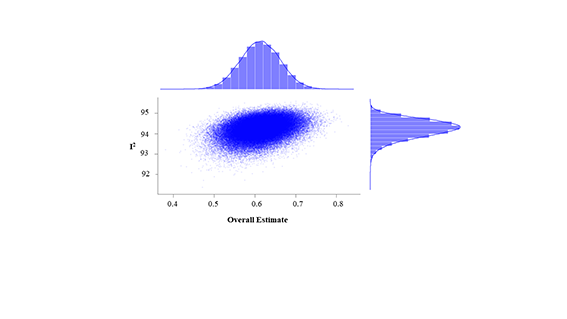

Supplement: Supplementary Figure 2 — The graphic display of study heterogeneity. [file Image_2.TIF]
