# Supplementary material for: A New Method to Address the Importance of Detoxified Enzyme in Insecticide Resistance – Meta-Analysis
Source: Front Physiol. 2022 Mar 2;13:818531. doi: 10.3389/fphys.2022.818531 (PMC8924616; doi:10.3389/fphys.2022.818531)
Supplement: Supplementary Data 5 — R code for meta-analyses. [file Data_Sheet_1.PDF]

```
#prepare the package used in meta-analysis####
library(readxl)
library(meta)
library(metafor)
library(dmetar)
library(dplyr)
library(pipeR)


##Read File####
Total.data.extraction <- read_excel("Table S1.xlsx",
                        sheet = "Total data extraction", col_types = c("text",
                                               "text", "text", "text", "text", "text",
                                               "text", "text", "text", "text", "text",
                                               "text", "numeric", "numeric", "numeric",
                                               "numeric", "numeric", "numeric","numeric"))



With.susceptible.data <- read_excel("Table S1.xlsx",
                        sheet = "With susceptible data", col_types = c("text",
                                               "text", "text", "text", "text", "text",
                                               "text", "text", "text", "text", "text",
                                               "text", "numeric", "numeric", "numeric",
                                               "numeric", "numeric", "numeric"))




Without.susceptible.data <- read_excel("Table S1.xlsx",
                         sheet = "Without susceptible data", col_types = c("text",
                                                  "text", "text", "text", "text", "text",
                                                  "text", "text", "text", "text", "text",
                                                  "text", "numeric", "numeric", "numeric",
                                                  "numeric", "numeric", "numeric",'numeric'))

yifan_wang <- Total.data.extraction
###paper numbers####
##how many studies in total and their author and year ##
unique(yifan_wang$Studies)%>>%as.vector()%>>% length()
unique(yifan_wang$Studies) %>>% as.vector() %>>% print()

##how many classes of insecticides intotal in this paper##
insecticide_class <- unique(yifan_wang$Insecticide_class) %>>% as.vector()
## how many studies in each of class of insecticide##
insecticide.class.number <- vector('list')
for(i in insecticide_class[1:12]){
  insecticide.class.number[i] <- subset(yifan_wang,Insecticide_class == i) %>% .[,1] %>% unique() %>% nrow() %>%
print()
}

##how many vector types intotal in this paper, and how may studies in each of type of vector##
vector_type <- unique(yifan_wang$vector) %>>% as.vector()
```

```
vectr.type.number <- vector('list')
for(i in vector_type){
  vectr.type.number[i] <- subset(yifan_wang,vector == i) %>% .[,1] %>% unique() %>% nrow() %>% print()
}

##how many species in medical vector in this paper
unique(medical.vector$Genus) %>>% as.vector()
##how many speceis in agricultural vector in this paper
unique(agricultural.vector$Genus) %>>% as.vector()


##how many paper in gene family under medical vetor group

medical.genefamily <- vector('list')
for (i in gene_family) {
  medical.genefamily[i] <- subset(medical.vector,Gene_function == i) %>% .[,1] %>% unique() %>% length() %>%
print()
}

##how many paper in gene family under agricultural vector group

agricultural.genefamily <- vector('list')
for (i in gene_family) {
  agricultural.genefamily[i] <- subset(agricultural.vector, Gene_function == i) %>% .[,1] %>% unique() %>% length()
}




##how many gene-family in total in this paper, and how many studies in each type of gene family##
gene_family <- unique(yifan_wang$Gene_function) %>>% as.vector()
gene.family.number <- vector('list')
for(i in gene_family){
  gene.family.number[i] <- subset(yifan_wang,Gene_function == i) %>% .[,1] %>% unique() %>% nrow() %>% print()
}

##how many p450 clade in this paper, and how many studies in each clade of P450##
p450_clade <- unique(yifan_wang$Clade) %>>% as.vector()
p450.clade.number <- vector('list')
for(i in p450_clade){
  p450.clade.number[i] <- subset(yifan_wang,Clade == i) %>% .[,1] %>% unique() %>% nrow() %>% print()
}


p450_clade_fixed <- unique(yifan_wang$Clade_fixed) %>>% as.vector()
p450.clade.fixed.number <- vector('list')
for(i in p450_clade_fixed){
  p450.clade.fixed.number[i] <- subset(yifan_wang,Clade_fixed == i) %>% .[,1] %>% unique() %>% nrow() %>%
print()
}
##how many genus in this paper, and how many studies in each of genus##
Genus <- unique(yifan_wang$Genus) %>>% as.vector()
genus.number <- vector('list')
for(i in Genus){
```

```
  genus.number[i] <- subset(yifan_wang,Genus == i) %>% .[,1] %>% unique() %>% nrow() %>% print()
}

###calculate the effect size for the studies with susceptible data by log-response ratio in metafor package####
effect.size.with.susceptible.data <- escalc(n1i = T_sample_size, m1i = T_mean, sd1i = T_sd,n2i = C_sample_size, m2i
= C_mean, sd2i = C_sd, data = With.susceptible.data, measure = "ROM")

Without.susceptible.data$yi <- Without.susceptible.data$`lnT-mean`

effect.size.whole <- merge(Without.susceptible.data, effect.size.with.susceptible.data, all = T)

###run the unweighted analysis with the whole data (0.1 been used as error) in metafor package####
whole_result.metafor.unweighted <- rma(vi = 0.1, yi = yi, ni=C_sample_size, data = effect.size.whole, slab =
paste(effect.size.whole$Studies,step=", "), method = "REML", weighted = F)
summary(whole_result.metafor.unweighted)
whole_result.metafor.unweighted$pval
exp(whole_result.metafor.unweighted$b)
exp(whole_result.metafor.unweighted$ci.lb)
exp(whole_result.metafor.unweighted$ci.ub)
###run the weighted analysis with the data including the sd of both susceptible group and resistance group####
With.susceptible.metafor.weighted <-rma(vi = vi, yi = yi, data = effect.size.with.susceptible.data, slab =
paste(effect.size.with.susceptible.data$Studies,step=", "), method = "REML")
summary(With.susceptible.metafor.weighted)
exp(With.susceptible.metafor.weighted$b)
exp(With.susceptible.metafor.weighted$ci.lb)
exp(With.susceptible.metafor.weighted$ci.ub)
###run the meta analysis for the studies which contains susceptible data in meta package ######
with.susceptible.meta <- metagen(
  TE = yi,
  seTE = vi,
  data = effect.size.with.susceptible.data,
  studlab =  paste(Studies,  step=", "),
  comb.fixed = F,
  comb.random = T,
  method.tau = "SJ",
  hakn = T,
  prediction = F,
  sm = "ROM",
  backtransf = F
)
summary(with.susceptible.meta)

##leave 1 out analysis for whole result unweighted analysis#####
unweighted.result.sensitivity <- leave1out(whole_result.metafor.unweighted)
unweighted.sensitivity.analysis <- data.frame(unweighted.result.sensitivity)

write.csv(unweighted.sensitivity.analysis," Sensitivity analysis for the whole unweighted meta-analysis.csv")
max(unweighted.sensitivity.analysis$estimate)
min(unweighted.sensitivity.analysis$estimate)

###leave 1 out analysis for with susceptible data weighted analysis####
With.susceptible.result.sensitivity <- leave1out(With.susceptible.metafor.weighted)
With.susceptible.sensitivity <- data.frame(With.susceptible.result.sensitivity)
```

```
write.csv(With.susceptible.sensitivity," Sensitivity analysis for the weighted meta-analysis.csv")
max(With.susceptible.sensitivity$estimate)
min(With.susceptible.sensitivity$estimate)

###change the format of the leave 1 out analysis####
Sensitivity_analysis_for_the_whole_unweighted_meta_analysis$`Confidence Interval`<- paste(
  '',
  round(S5_Sensitivity_analysis_for_the_whole_unweighted_meta_analysis$`lower bounds of confidence interval`,3),
  step = ' - ',
  round(S5_Sensitivity_analysis_for_the_whole_unweighted_meta_analysis$`upper-bounds of confidence interval`,3)
)
write.csv(S5_Sensitivity_analysis_for_the_whole_unweighted_meta_analysis, 'sensitivity analysis for the whole
unweighted meta analysis.csv')

Sensitivity_analysis_for_the_weighted_meta_analysis$`Confidence Interval`<- paste(
  '',
  round(S6_Sensitivity_analysis_for_the_weighted_meta_analysis$`lower bounds of confidence interval`,3),
  step = ' - ',
  round(S6_Sensitivity_analysis_for_the_weighted_meta_analysis$`upper-bounds of confidence interval`,3)
)
write.csv(S6_Sensitivity_analysis_for_the_weighted_meta_analysis, 'sensitivity analysis for the weighted meta
analysis.csv')

###funnel plot and publication bias######
jpeg(filename = "funnel plot of with susceptible data weighted analysis in metafor.jpeg", width = 1600, height = 900,
quality = 2000, bg="white")
viz_funnel(With.susceptible.metafor.weighted)
dev.off()

regtest(With.susceptible.metafor.weighted)
eggers.test(with.susceptible.meta)

###forest plot of whole unweighted analysis####
jpeg(filename = " Unweighted meta analytical forest plot.jpeg", width = 1100, height = 6000, quality = 2000,
bg="white")
forest(whole_result.metafor.unweighted,xlim=c(-50,10), pch = 19, psize = 1,efac=0.1,ylim = c(0,409),
at=log(c(0.01,0.1,1,10)),
      ilab = cbind(effect.size.whole$Gene, effect.size.whole$Species, effect.size.whole$Insecticide,
effect.size.whole$vector),
      fonts = 'serif',
      ilab.xpos = c(-40,-30,-20,-10),mlab = "",cex=0.75,xlab = '')
op <- par(cex=1, font=2)
text(c(-48,-40,-30,-20,-10,0,7),     409, c("Study",'Gene','Species','Insecticide','Vector','Log Ratio of Means','ROM &
95%CI'))
par(op)
text(-50, -1, pos=4,cex=1, font=2, bquote(paste("RE Model for unweighted analysis of whole studies (Q = ",
                                        .(formatC(whole_result.metafor.unweighted$QE, digits=2, format="f")), ", df = ", .
(whole_result.metafor.unweighted$k - whole_result.metafor.unweighted$p),
                                        ", p = ", .(formatC(whole_result.metafor.unweighted$QEp, digits=2, format="f")), "; ",
I^2, " = ",
                                        .(formatC(whole_result.metafor.unweighted$I2, digits=1, format="f")), "%)")))
dev.off()

png(filename = " Unweighted meta analytical forest plot.png", width = 1500, height = 7000, pointsize = 20, bg="white")
```

```
forest(whole_result.metafor.unweighted,xlim=c(-50,10), pch = 19, psize = 1,efac=0.1,ylim = c(0,409), 
at=log(c(0.01,0.1,1,10)),
       ilab = cbind(effect.size.whole$Gene, effect.size.whole$Species, effect.size.whole$Insecticide, 
effect.size.whole$vector),
       fonts = 'serif',
       ilab.xpos = c(-40,-30,-20,-10),mlab = "",cex=0.75,xlab = '')
op <- par(cex=1, font=2)
text(c(-48,-40,-30,-20,-10,0,7),     409, c("Study",'Gene','Species','Insecticide','Vector','Log Ratio of Means','ROM & 
95%CI'))
par(op)
text(-50, -1, pos=4,cex=1, font=2, bquote(paste("RE Model for unweighted analysis of whole studies (Q = ",
                                        .(formatC(whole_result.metafor.unweighted$QE, digits=2, format="f")), ", df = ", .
(whole_result.metafor.unweighted$k - whole_result.metafor.unweighted$p),
                                        ", p = ", .(formatC(whole_result.metafor.unweighted$QEp, digits=2, format="f")), "; ", 
I^2, " = ",
                                        .(formatC(whole_result.metafor.unweighted$I2, digits=1, format="f")), "%)")))
dev.off()
##forest plot of weighted analysis####
jpeg(filename = " Weighted meta analytical forest plot.jpeg", width = 1100, height = 6000, quality = 2000, bg="white")
forest(With.susceptible.metafor.weighted,xlim=c(-50,10), pch = 19, psize = 1,efac=0.1,ylim = c(0,287), 
at=log(c(0.01,0.1,1,10)),
       ilab = cbind(effect.size.whole$Gene, effect.size.whole$Species, effect.size.whole$Insecticide, 
effect.size.whole$vector),
       fonts = 'serif',
       ilab.xpos = c(-40,-30,-20,-10),mlab = "",cex=0.75,xlab = '')
op <- par(cex=1, font=2)
text(c(-48,-40,-30,-20,-10,0,7),     287, c("Study",'Gene','Species','Insecticide','Vector','Log Ratio of Means','ROM & 
95%CI'))
par(op)
text(-50, -1, pos=4,cex=1, font=2, bquote(paste("RE Model for unweighted analysis of whole studies (Q = ",
                                        .(formatC(With.susceptible.metafor.weighted$QE, digits=2, format="f")), ", df = ", .
(With.susceptible.metafor.weighted$k - With.susceptible.metafor.weighted$p),
                                        ", p = ", .(formatC(With.susceptible.metafor.weighted$QEp, digits=2, format="f")), "; ", 
I^2, " = ",
                                        .(formatC(With.susceptible.metafor.weighted$I2, digits=1, format="f")), "%)")))
dev.off()

png(filename = " Weighted meta analytical forest plot.png", width = 1500, height = 6000, pointsize = 20, bg="white")
forest(With.susceptible.metafor.weighted,xlim=c(-50,10), pch = 19, psize = 1,efac=0.1,ylim = c(0,287), 
at=log(c(0.01,0.1,1,10)),
       ilab = cbind(effect.size.whole$Gene, effect.size.whole$Species, effect.size.whole$Insecticide, 
effect.size.whole$vector),
       fonts = 'serif',
       ilab.xpos = c(-40,-30,-20,-10),mlab = "",cex=0.75,xlab = '')
op <- par(cex=1, font=2)
text(c(-48,-40,-30,-20,-10,0,7),     287, c("Study",'Gene','Species','Insecticide','Vector','Log Ratio of Means','ROM & 
95%CI'))
par(op)
text(-50, -1, pos=4,cex=1, font=2, bquote(paste("RE Model for unweighted analysis of whole studies (Q = ",
                                        .(formatC(With.susceptible.metafor.weighted$QE, digits=2, format="f")), ", df = ", .
(With.susceptible.metafor.weighted$k - With.susceptible.metafor.weighted$p),
                                        ", p = ", .(formatC(With.susceptible.metafor.weighted$QEp, digits=2, format="f")), "; ", 
I^2, " = ",
                                        .(formatC(With.susceptible.metafor.weighted$I2, digits=1, format="f")), "%)")))
```

```
dev.off()
###Run subgroup analysis for whole effect size in unweighted analysis####
##agricultural##
agricultural.vector <- subset(effect.size.whole, vector == "Agricultural pest")
medical.vector <- subset(effect.size.whole, vector == "Medical pest")


agricultural <- rma(vi = 0.1, yi = yi, data = agricultural.vector, slab = paste(Studies,step=", "), method = "REML",
weighted = F)
summary(agricultural)

agricultural.gene.family <- rma(vi = 0.1, yi = yi, data = agricultural.vector, slab = paste(Studies,step=", "), method =
"REML", weighted = F, mods = ~Gene_function-1)
summary(agricultural.gene.family)

agricultural.gene.family$bylevs <- row.names(agricultural.gene.family$beta)
agricultural.gene.family.effect.size <- data.frame(agricultural.gene.family$bylevs, agricultural.gene.family$beta[,1],
agricultural.gene.family$ci.lb, agricultural.gene.family$ci.ub, agricultural.gene.family$pval)
colnames(agricultural.gene.family.effect.size) <- c('bylevs','b','ci.lb', 'ci.ub','p val')

##medical##
medical <- rma(vi = 0.1, yi = yi, data = medical.vector, slab = paste(Studies,step=", "), method = "REML", weighted =
F)
summary(medical)

medical.gene.family <- rma(vi = 0.1, yi = yi, data = medical.vector, slab = paste(Studies,step=", "), method = "REML",
weighted = F,mods = ~Gene_function-1)
summary(medical.gene.family)

medical.gene.family$bylevs <- row.names(medical.gene.family$beta)
medical.gene.family.effect.size <- data.frame(medical.gene.family$bylevs, medical.gene.family$beta[,1],
medical.gene.family$ci.lb, medical.gene.family$ci.ub, medical.gene.family$pval)
colnames(medical.gene.family.effect.size) <- c('bylevs','b','ci.lb', 'ci.ub','p val')

##P450##
p450 <- subset(effect.size.whole, Gene_function == 'p450')

p450.subgroup <- rma(vi = 0.1, yi = yi, data = p450, slab = paste(Studies,step=", "), method = "REML", weighted = F,
mods = ~Clade-1)
summary(p450.subgroup)

p450.subgroup$bylevs <- row.names(p450.subgroup$beta)
p450.subgroup.effect.size <- data.frame(p450.subgroup$bylevs, p450.subgroup$beta[,1], p450.subgroup$ci.lb,
p450.subgroup$ci.ub, p450.subgroup$pval)
colnames(p450.subgroup.effect.size) <- c('bylevs','b','ci.lb', 'ci.ub','p val')

##insecticide class##
insecticide.class.subgroup <- rma(vi = 0.1, yi = yi, data = effect.size.whole, slab = paste(Studies,step=", "), method =
"REML", weighted = F,mods = ~Insecticide_class-1)
summary(insecticide.class.subgroup)

insecticide.class.subgroup$bylevs <- row.names(insecticide.class.subgroup$beta)
insecticide.class.subgroup.effect.size <- data.frame(insecticide.class.subgroup$bylevs,
insecticide.class.subgroup$beta[,1], insecticide.class.subgroup$ci.lb, insecticide.class.subgroup$ci.ub,
```

```
insecticide.class.subgroup$pval)
colnames(insecticide.class.subgroup.effect.size) <- c('bylevs','b','ci.lb', 'ci.ub','p val')

##genus##
genus.subgroup <- rma(vi = 0.1, yi = yi, data = effect.size.whole, slab = paste(Studies,step=", "), method = "REML",
weighted = F,mods = ~Genus-1)
summary(genus.subgroup)

genus.subgroup$bylevs <- row.names(genus.subgroup$beta)
genus.subgroup.effect.size <- data.frame(genus.subgroup$bylevs, genus.subgroup$beta[,1], genus.subgroup$ci.lb,
genus.subgroup$ci.ub, genus.subgroup$pval)
colnames(genus.subgroup.effect.size) <- c('bylevs','b','ci.lb', 'ci.ub','p val')

##vector##
vector.subgroup <- rma(vi = 0.1, yi = yi, data = effect.size.whole, slab = paste(Studies,step=", "), method = "REML",
weighted = F,mods = ~vector-1)
summary(vector.subgroup)

vector.subgroup$bylevs <- row.names(vector.subgroup$beta)
vector.subgroup.effect.size <- data.frame(vector.subgroup$bylevs, vector.subgroup$beta[,1], vector.subgroup$ci.lb,
vector.subgroup$ci.ub, vector.subgroup$pval)
colnames(vector.subgroup.effect.size) <- c('bylevs','b','ci.lb', 'ci.ub','p val')

##gene family##
gene.family.subgroup <- rma(vi = 0.1, yi = yi, data = effect.size.whole, slab = paste(Studies,step=", "), method =
"REML", weighted = F,mods = ~Gene_function-1)
summary(gene.family.subgroup)

gene.family.subgroup$bylevs <- row.names(gene.family.subgroup$beta)
gene.family.subgroup.effect.size <- data.frame(gene.family.subgroup$bylevs, gene.family.subgroup$beta[,1],
gene.family.subgroup$ci.lb, gene.family.subgroup$ci.ub, gene.family.subgroup$pval)
colnames(gene.family.subgroup.effect.size) <- c('bylevs','b','ci.lb', 'ci.ub','p val')

subgroup.effect.size.plot <- bind_rows(gene.family.subgroup.effect.size,
                                insecticide.class.subgroup.effect.size,
                                genus.subgroup.effect.size,
                                vector.subgroup.effect.size,
                                p450.subgroup.effect.size,
                                agricultural.gene.family.effect.size,
                                medical.gene.family.effect.size)

subgroup.effect.size.plot <- data.frame(subgroup.effect.size.plot)
duplicated(subgroup.effect.size.plot)

write.csv(subgroup.effect.size.plot, 'subgroup effect size plot.csv')
###change the format of the subgrop effect size table in unweighted analysis####
subgroup_effect_size_plot <- read_excel("subgroup effect size plot.xlsx",
                                 +     col_types = c("text", "text", "numeric",
                                                +         "numeric", "numeric", "numeric",
                                                +         "numeric", "numeric", "numeric"))


UPCI <- subgroup_effect_size_plot$ci.ub %>% round(3)
LOCI <- subgroup_effect_size_plot$ci.lb %>% round(3)
```

```
subgroup_effect_size_plot$`Effect Size` <- subgroup_effect_size_plot$`effect size` %>% round(3)
subgroup_effect_size_plot$`95% Confidence Interval` <- paste('',LOCI, step = ' - ', UPCI)


subgroup_effect_size_plot$`Linear CILO` <- exp(subgroup_effect_size_plot$ci.lb)%>% round(3)
subgroup_effect_size_plot$`Linear CIUP` <- exp(subgroup_effect_size_plot$ci.ub) %>% round(3)
subgroup_effect_size_plot$`95% Linearlized Confidence Interval` <- paste(
  '',
  subgroup_effect_size_plot$`Linear CILO`,
  ' - ',
  subgroup_effect_size_plot$`Linear CIUP`
)

write.csv(subgroup_effect_size_plot, 'subgroup effect size formatted.csv')

###find the pvalue of subgroup analysis########
##find the pvalue of gene family##
gene.function <- effect.size.whole[order(effect.size.whole$Gene_function,decreasing = T,na.last = T),]

gene.function$Gene_function <- relevel(factor(gene.function$Gene_function),ref = 'GST')
whole_result.metafor.gene.function.GST <- rma(vi=0.1,yi=yi, sd2i=C_sd, data=gene.function, measure = "ROM",
                                      slab = paste(Studies, step='', ''), method = "REML", mods = ~Gene_function)

summary(whole_result.metafor.gene.function.GST)
whole_result.metafor.gene.function.GST$pval%>% round(.,3)

gene.function$Gene_function <- relevel(factor(gene.function$Gene_function),ref = 'p450')
whole_result.metafor.gene.function.p450 <- rma(vi=0.1,yi=yi, data=gene.function, measure = "ROM",
                                      slab = paste(Studies, step='', ''), method = "REML", mods = ~Gene_function)
summary(whole_result.metafor.gene.function.p450)
whole_result.metafor.gene.function.p450$pval%>% round(.,3)


####p value of agricultural and medical vector###
whole_result.metafor.vector <- rma(vi=0.1, yi=yi, data=effect.size.whole, measure = "ROM",
                              slab = paste(Studies, step='', ''), method = "REML", mods = ~vector)
summary(whole_result.metafor.vector)
whole_result.metafor.vector$pval%>%round(.,3)

###pcurve######

jpeg(filename = " P-curve of the weighted meta-analysis.jpeg", width = 800, height = 600, quality = 10000)
pcurve(with.susceptible.meta)
dev.off()

png(filename = " P-curve of the weighted meta-analysis.png", width = 800, height = 600, units = 'px',pointsize = 16)
pcurve(with.susceptible.meta)
dev.off()
###Graphic Display of study Heterogeneity ####
dat.gosh <- gosh(whole_result.metafor.unweighted)
summary(dat.gosh)
gosh.diagnostics(dat.gosh)
```

```
jpeg(filename =" The Graphic Display of Study Heterogeneity.jpeg",width = 1600, height = 900, quality = 2000, res =
300)
plot(dat.gosh, alpha = 0.1, col = "blue",fonts = 'serif')
dev.off()

png(filename =" The Graphic Display of Study Heterogeneity.png",width = 1600, height = 900, pointsize = 30)
plot(dat.gosh, alpha = 0.1, col = "blue",fonts = 'serif')
dev.off()
```
